# Successful resuscitation and multidisciplinary management of penetrating brain injury caused by tire explosion: A case report

**DOI:** 10.1097/MD.0000000000032048

**Published:** 2022-11-25

**Authors:** Haozhan Wang, Hao Chen, Changtong Liu, Long Yuan, Yonggang Bao, Guodong Zhao, Dengqin Wang, Guohong Song

**Affiliations:** a Department of Clinical Medicine, Jining Medical University, Jining, Shandong Province, China; b Department of Neurosurgery, Affiliated Hospital of Jining Medical University, Jining, Shandong, China.

**Keywords:** blast injury, combined multidisciplinary surgery, intracranial foreign body, penetrating brain injury

## Abstract

Penetrating brain injury (PBI) is a rare trauma that presents as a difficult and serious surgical emergency for neurosurgeons in clinical practice. Our patient was admitted with a PBI caused by a tire explosion, which is an extremely rare cause of injury.

**Patient concerns:**

We report a case of a 28-year-old male patient who suffered a PBI when a tire exploded while it was being inflated with a high-pressure air pump.

**Diagnoses:**

The patient was diagnosed with PBI presenting with multiple comminuted skull fractures, massive bone fragments with foreign bodies penetrating the underlying brain tissue of the top right frontal bone, multiple cerebral contusions, and intracranial hematoma.

**Interventions:**

Emergency combined multidisciplinary surgery was performed for the removal of the fragmented bone pieces, hematoma, and foreign bodies; decompression of the debridement flap; reconstruction of the anterior skull base; and repair of the dura mater.

**outcomes:**

The patient was successfully resuscitated and discharged 1 month later and is now recovering well.

**Lessons:**

Patients with PBI are critically ill. Therefore, timely, targeted examinations and appropriate multidisciplinary interventions through a green channel play a key role in assessing the condition, developing protocols, and preventing complications.

## 1. Introduction

Penetrating brain injury (PBI) is a lesion caused when a foreign body or bone fragment pierces brain tissue.^[1]^ PBI is mostly caused by the impact of ballistic, high-speed moving objects and has complex injury mechanisms and high post-injury mortality. PBI caused by non-ballistic, low-velocity objects is rare, and it is usually caused by violence, accidents, or even suicide attempts.^[2]^ Our patient was admitted with a PBI caused by a tire explosion, which is an extremely rare cause of injury.

## 2. Case report

### 2.1. Present medical history

The patient was a 28-year-old male who was admitted to the Affiliated Hospital of Jining Medical College on February 4, 2022 for “bleeding and pain in the head and eyes for 2 h after trauma.” The trauma occurred due to a tire explosion that occurred while the patient was using a high-pressure air pump to inflate the tire. The explosion injured the patient’s head, which caused immediate head and eye pain, along with bleeding from the head wound and brain tissue spillage, impaired consciousness without coma, chest tightness, palpitation, dyspnea, and dizziness.

### 2.2. Past history

The patient was in good health and had no history of hypertension, diabetes, coronary heart disease, or similar diseases.

### 2.3. Physical examination

Vital signs were as follows: temperature, 36.7°C; pulse, 49 beats/min; respiration, 20 breaths/min; and blood pressure, 129/87 mm Hg. Physical examination revealed an open wound at 0.5 cm on the right side of the patient’s glabella with brain tissue spillage. The right eye was fixed with dressing, whereas pupil and visual acuity were not examined. The muscle strength of the limbs was grade 5, with normal muscle tone.

### 2.4. Auxiliary examination

**Figure 1.**
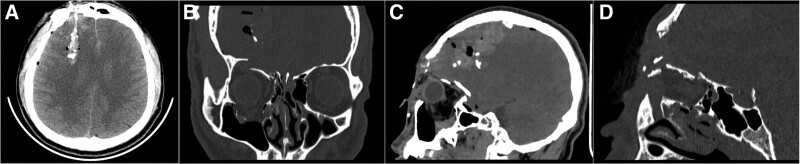
Preoperative CT images. (A) Axial slice showing the location of the skull break, injury tract, and foreign body. (B) Coronal image revealing the fractures of the superior and inferior walls of the orbit, as well as the downward collapse of the eye and its contents. (C) Sagittal image demonstrating air and multiple fragments of bone inside the brain, local hematoma complication, and fractures of the superior orbital wall and anterior skull base. (D) Sagittal reconstructed CT image with the break located in the right frontal sinus, superior orbital wall, and anterior skull base. CT = computed tomography.

Cranial computed tomography (CT) examination was performed in the emergency room after admission (Fig. 1). The findings were as follows: right frontal lobe cerebral contusion (cerebral hematoma formation); traumatic subarachnoid hemorrhage; multiple intracranial gas accumulation with free fragmented bone pieces; comminuted fracture of the right frontal bone (involving the right frontal sinus, upper and lower orbital wall, and medial wall); hemorrhagic effusion in the bilateral frontal sinus, septal sinus, and right maxillary sinus; and right ethmoidal labyrinth fracture.

Admission diagnosis was as follows: PBI, multiple cerebral contusions with intracranial hematoma, intracranial pneumatization with multiple foreign bodies, multiple comminuted skull fractures, maxillofacial blast injury, intraorbital tissue loss and injury, medial orbital skin contusion, ulnar styloid fracture, and multiple fractures of the fingers.

Due to the PBI and the complexity of the patient’s condition, treatment by a single department was not sufficient. Therefore, a multidisciplinary team consultation mechanism was immediately launched, which involved the urgent convening of experts from relevant departments, such as neurosurgery, ophthalmology, otorhinolaryngology, maxillofacial surgery, traumatology and orthopedics, and critical care medicine, to facilitate a complete discussion and formulate a detailed treatment plan. The main difficulties were identified to include 4 aspects. The first aspect was the lack of experience. This was because most patients with cranial penetrating injuries die on the spot. Furthermore, Chinese neurosurgeons are less exposed to such conditions, and their surgical treatment lacks guidelines and norms regarding this injury. Moreover, this condition requires extremely demanding skills from the operator. The second aspect involved the difficulty of debridement. This was because the patient had an open wound with multiple surrounding comminuted fractures, and the broken bone fragments had penetrated into the right frontoparietal brain tissue at about 10 cm. Moreover, the location of the injury was deep and extensive (the size of the injury area was about 15 cm × 10 cm × 6 cm), while the size of the intracranial foreign body was different. This posed a great challenge for thorough debridement. The third difficulty was that the anterior skull base structure was severely damaged. Therefore, the entire right regions of the anterior skull base dura and plate, supraorbital wall, and frontal sinus had to be reconstructed. However, the surrounding area was full of important nerves, blood vessels, and organs, with even the slightest damage leading to the failure of the operation. The fourth complication was that postoperative recovery was extremely difficult. Postoperative recovery not only involves preventing and treating serious complications, such as cerebral hemorrhage, cerebral infarction, cerebral edema, epilepsy, intracranial and extracranial infections, and pulmonary infections, but also helping patients recover their neurological function and vision.

The multidisciplinary team agreed that only early combined multidisciplinary surgery could save the patient’s life and devised a plan accordingly. First, the operating room and anesthesia department were notified to make adequate preparations. Additionally, the cardiopulmonary team was notified to be available to provide the necessary resuscitation measures. Subsequently, specialists from all departments were convened in the operating room. Next, we communicated with the family again before the surgery. Finally, all specialists agreed that adequate preparation had been made to begin exploration, cranial repair, and foreign body removal.

In the operating room, a comminuted multiple fracture fragment debridement with enlargement of the original wound, subdural hematoma and intracerebral hematoma removal, anterior skull base reconstruction, dural repair, and debridement suture were performed under compound anesthesia with static suction. The surgery was performed by a neurosurgeon using the following procedure. First, a curved incision was made along the wound to enlarge it, followed by the discovery of comminuted orbital bone and superior frontal sinus bone about 4 cm × 3 cm in size. The fragmented bone penetrated about 10 cm into the brain tissue underlying the top frontal bone. Additionally, a small subdural hematoma (about 3 cm × 3 cm × 3 cm) and intracerebral hematoma (about 10 cm × 3 cm × 3 cm) were revealed. The wound was repeatedly flushed with iodophor disinfectant and saline, and the fracture fragments, hair, and subcutaneous tissue were removed. Next, 1 hole at the supraorbital margin and one 2-cm hole posterior to the coronal suture were drilled, and a bone flap (approximately 10 cm × 3 cm) was removed. Furthermore, 2 ruptures (approximately 3 cm × 3 cm and 2 cm × 3 cm) were found in the dura mater, with cerebrospinal fluid spillage. The dura mater was suspended, and the fragmented brain tissue was carefully removed to find and remove the fracture fragments in the brain. Subsequently, hemostatic gauze was used to stop the bleeding, and a large amount of saline was flushed until the region was clear and no active bleeding was visible. Next, bone cement was used to seal the frontal sinus and reconstruct the anterior skull base, and fascia was utilized to repair and tightly suture the torn dura mater. The operation lasted nearly 12 hours, with 100 mL of intraoperative blood loss (however, no blood transfusion was required), 5400 mL of fluid transfusion, and 1800 mL of urine volume.

After surgery, the patient was transferred to an intensive care unit, where he was intubated and ventilated and underwent close monitoring of his vital signs. Due to the severe trauma and wound contamination, the patient was treated postoperatively mainly with anticoagulation and anti-infection therapy as well as prophylaxis for epilepsy.

**Figure 2.**
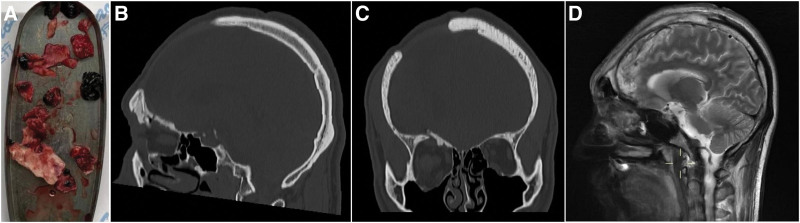
(A) Intraoperative removal of the bone fragments, hematoma, and tissue. (B) Postoperative sagittal reconstructed CT image showing the removal of bone fragments and hematoma. (C) Postoperative coronal reconstructed CT image demonstrating the reconstruction of the anterior skull base and supraorbital wall. (D) MRI sagittal image at postoperative 2 weeks revealing good recovery of the operated area. CT = computed tomography, MRI = magnetic resonance imaging.

**Figure 3.**
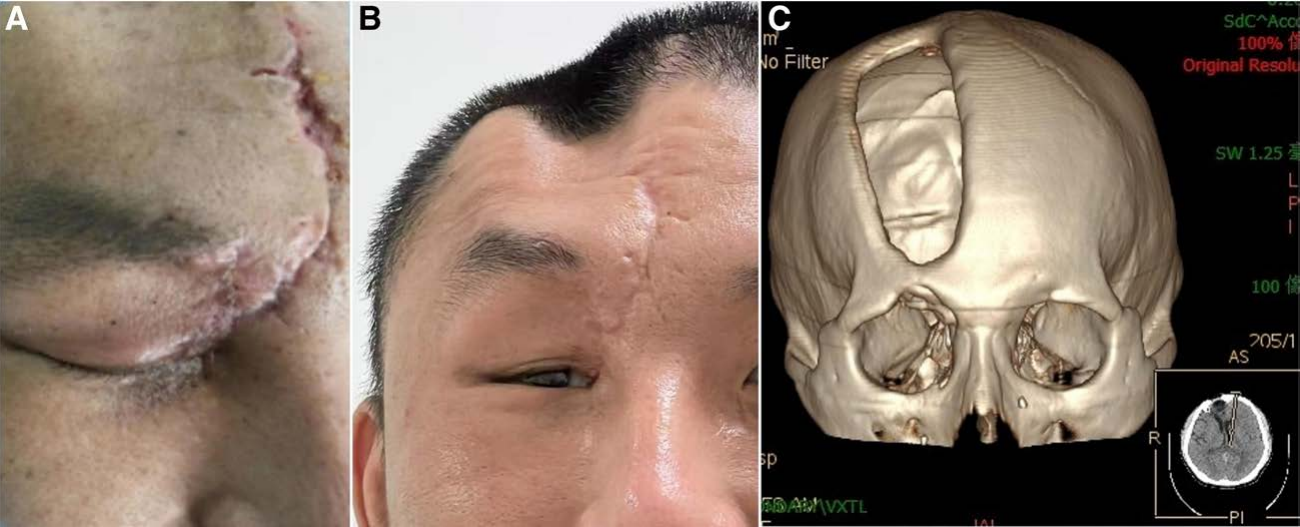
(A) Wound healing of the patient at the time of discharge, with inability to open the injured right eye. (B) At postoperative 6 months, the patient recovered well. The injured right eye could be opened, and the eye movement was normal; however, the eye fissure was slightly reduced. (C) Cranial reconstruction images at postoperative 6 months showing good cranial recovery.

After 1 month of treatment, several imaging examinations demonstrated the patient’s recovery (Fig. 2). After a normal assessment of his condition, the patient was discharged on March 8, 2022, with his right eyelid in a closed state and difficulty opening his eyes at the time of discharge. At the 6-month postoperative review (Fig. 3), the patient exhibited good skull base, cranial, and dural reconstruction and good recovery of neurological function and ocular visual acuity and movement, except for a smaller eye fissure on the injured side. At present, the patient’s eye function and eyelid movement have returned to normal, and the wound is healing well with no sequelae.

## 3. Discussion

Tires contain high-pressure air, and similar to an improvised explosive device, the main injury mechanism is the injury caused by the high-energy shock wave formed by the pressure difference generated by the explosion, while the secondary injury is inflicted by the fragments and surrounding objects hitting and penetrating the human body after the explosion.^[3]^ In these explosions, the degree of injury depends on the explosion energy and the distance from the center of the explosion.^[4]^ Previous literature has reported the serious hazard of tire explosions to humans, and in a retrospective study by Suruda et al, 143 of 694 people injured by tire explosions died, with a 78% mortality rate from head injuries.^[5]^ Sheperd et al analyzed and summarized the injury pattern of tire explosions and found that the face (58%) and skull (33%) were the common injury sites, with the most severe penetrating injuries to the head.^[6]^ Over the years, the increased safety awareness and advances in related equipment have reduced the number of people being injured by tire blasts, resulting in the related literature becoming even rarer. Our patient was in close proximity to a truck tire when the tire exploded during inflation, thus causing severe PBI.

PBIs are rare and have a mortality rate of 23% to 93%, which increases to 87% to 100% when vital nerves are injured.^[7–9]^ Therefore, most patients with PBI die at the accident scene. Harrington et al found that patients with PBI and having wounds in the orbital apex are not usually injured in vital vascular areas,^[10]^ owing to which, these patients are able to arrive at hospitals for treatment. However, even after hospital treatment, half of the patients may still not survive, while those who survive experience varying degrees of sequelae.^[11]^ Thus, the treatment of patients with PBI is a great challenge to hospital management and demanding of the physician skills.

For PBI diagnosis, non-enhanced CT is preferred owing to its speed, ubiquity, and high sensitivity in detecting entry and exit wounds, fractures, metallic foreign bodies, hemorrhage, and occupancy effects.^[12]^ Therefore, we activated a green channel immediately after the patient’s admission and completed the CT examination in the shortest possible time, which provided us with the basis for understanding the patient’s condition and determining the surgical plan.

The CT results indicated that the patient’s injury was serious and emergency surgery was necessary to save his life. However, no relevant guidelines and norms are present in China or internationally, with most of the consensuses lacking sufficient practical evidence for support. The fourth edition of the U.S. Guidelines for the Management of Severe Traumatic Brain Injury suggests that the management and treatment of patients with traumatic brain injury require the use of a comprehensive approach, with no generalized treatment since patients usually have different injuries.^[13]^ Therefore, PBI surgery must be performed in the context of the patient’s specific condition and according to the relevant rules. Surgical treatment generally includes debridement, evacuation of hematomas, decompressive craniectomy, dural repair, and stereotaxic technique.^[14]^

The key to our surgery was the thorough removal of foreign bodies and hematomas. We performed the intracranial removal of dozens of foreign bodies of different sizes (ranging from 2 mm to 2 cm), which would have led to intracranial infection and even death. Additionally, the reconstruction of the skull base and dura mater is very difficult, and the main objectives of skull base reconstruction include restoring the structural support of the skull and orbit, separating the central nervous system from the respiratory and digestive tracts, reducing the volume of the dead space, and repairing the 3-dimensional appearance of the face and skull.^[15]^ We applied only bone cement to repair the skull base and orbital wall, with no application of artificial materials, such as artificial dura and titanium mesh. We sutured dozens of stitches during the anterior skull base repair of the dura, and each stitch greatly tested the operator’s skill because of the extremely large number of important surrounding structures.

Ultimately, we were able to complete the surgery with multidisciplinary efforts; however, the prevention and treatment of various postoperative complications were also quite problematic. Infection is a common complication of PBI, manifesting mainly as local wound infection, meningitis, or brain abscess.^[16]^ In this context, available literature recommend the early initiation of broad-spectrum antibiotic therapy in all cases of PBI.^[17]^ Additionally, since no uniform standards regarding the timing of antibiotic administration and the length of antibiotic treatment exist, the physician is required to administer relevant treatment taking into account the patient’s condition and clinical experience. Furthermore, seizure prevention is another important aspect. In a retrospective analysis by Loggini et al, patients with PBI were found to have the highest incidence of seizures within the first 7 days after the injury,^[18]^ and phenytoin sodium is recommended to reduce the incidence of early post-traumatic seizures.^[13]^

Magnetic resonance imaging is mostly used for early postoperative assessment of secondary brain changes because it is time consuming and contraindicated in patients with metallic foreign bodies.^[19,20]^ After the patient was stabilized at 2 weeks postoperatively, we evaluated the patient’s brain injury, cerebrovascular injury, and cerebral edema using plain cranial magnetic resonance imaging and MR angiography and found that the patient had recovered well.

After 1 month of multidisciplinary management, the patient successfully passed the risk period for recurrent cerebral hemorrhage, cerebral infarction, cerebral edema, epilepsy, intracranial and extracranial infections, pulmonary infections, and other complications, and he gradually recovered neurological function, eye vision, and oculomotor function. At the 6-month postoperative follow-up, the patient’s general condition as well as somatic and neurological functions returned to normal, with only mild changes in the affected eye fissure.

In summary, the successful treatment of our patient provides a valuable reference for similar patients. During emergency treatment of PBI, maintenance of the airway, adequate ventilation, and appropriate treatment of hypoxia and hypotension can effectively prevent secondary brain injury. Additionally, the usefulness of rapid transfer to a tertiary trauma center, close intensive care, rapid examination through a green channel to clarify the diagnosis, and reasonable surgical intervention by a multidisciplinary team were all valuable lessons learned from this study.

## Author contributions

**Formal analysis:** Hao Chen.

**Funding acquisition:** Guohong Song.

**Methodology:** Yonggang Bao.

**Project administration:** Guohong Song.

**Resources:** Guodong Zhao, Guohong Song.

**Supervision:** Changtong Liu.

**Validation:** Guodong Zhao.

**Visualization:** Yonggang Bao.

**Writing – original draft:** Haozhan Wang.

**Writing – review & editing:** Haozhan Wang, Hao Chen, Long Yuan, Dengqin Wang.
